# Binding to Na^+^/H^+^ exchanger regulatory factor 2 (NHERF2) affects trafficking and function of the enteropathogenic *Escherichia coli* type III secretion system effectors Map, EspI and NleH

**DOI:** 10.1111/j.1462-5822.2010.01503.x

**Published:** 2010-08-03

**Authors:** Eric Martinez, Gunnar N Schroeder, Cedric N Berger, Sau Fung Lee, Keith S Robinson, Luminita Badea, Nandi Simpson, Randy A Hall, Elizabeth L Hartland, Valerie F Crepin, Gad Frankel

**Affiliations:** 1 Centre for Molecular Microbiology and Infection, Imperial College LondonUK; 2 Department of Microbiology and Immunology, University of MelbourneMelbourne, Vic. 3010, Australia; 3 Department of Pharmacology, Emory University School of MedicineAtlanta, GA 30322, USA

## Abstract

Enteropathogenic *Escherichia coli* (EPEC) strains are diarrhoeal pathogens that use a type III secretion system to translocate effector proteins into host cells in order to colonize and multiply in the human gut. Map, EspI and NleH1 are conserved EPEC effectors that possess a C-terminal class I PSD-95/Disc Large/ZO-1 (PDZ)-binding motif. Using a PDZ array screen we identified Na^+^/H^+^ exchanger regulatory factor 2 (NHERF2), a scaffold protein involved in tethering and recycling ion channels in polarized epithelia that contains two PDZ domains, as a common target of Map, EspI and NleH1. Using recombinant proteins and co-immunoprecipitation we confirmed that NHERF2 binds each of the effectors. We generated a HeLa cell line stably expressing HA-tagged NHERF2 and found that Map, EspI and NleH1 colocalize and interact with intracellular NHERF2 via their C-terminal PDZ-binding motif. Overexpression of NHERF2 enhanced the formation and persistence of Map-induced filopodia, accelerated the trafficking of EspI to the Golgi and diminished the anti-apoptotic activity of NleH1. The binding of multiple T3SS effectors to a single scaffold protein is unique. Our data suggest that NHERF2 may act as a plasma membrane sorting site, providing a novel regulatory mechanism to control the intracellular spatial and temporal effector protein activity.

## Introduction

Enteropathogenic *Escherichia coli* (EPEC) strains are Gram-negative pathogenic bacteria frequently associated with infantile diarrhoea in non-industrial countries (Chen and Frankel, 2005; Croxen and Finlay, 2010). During the course of infection, EPEC adheres intimately to the surface of enterocytes and provokes microvillus effacement, leading to the formation of characteristic attaching and effacing (A/E) lesions (Knutton *et al*., 1987). The ability to form A/E lesions is associated with the LEE pathogenicity island (McDaniel *et al*., 1995), which encodes gene regulators, the adhesin intimin, a type III secretion system (T3SS), chaperones and effector proteins (Garmendia *et al*., 2005). In addition, EPEC strains express a diverse number of non-LEE-encoded effectors (Iguchi *et al*., 2009).

Following translocation, the effector proteins are targeted to different cellular compartments and subvert numerous cellular processes to sustain colonization and multiplication (Garmendia *et al*., 2005). Map, EspI (also known as NleA) and NleH1 are conserved effectors and major EPEC virulence factors (Simpson *et al*., 2006; Hemrajani *et al*., 2008; Lee *et al*., 2008). NleH1 inhibits NF-κB-dependent transcription through an N-terminal interaction with ribosomal protein S3 (RPS3) (Gao *et al*., 2009) and prevents apoptosis through a C-terminal interaction with Bax inhibitor-1 (Hemrajani *et al*., 2010). EspI is targeted to the Golgi apparatus where it interacts with the Sec23/24 complex and inhibits COPII-mediated vesicular transport between the endoplasmic reticulum and the Golgi apparatus (Kim *et al*., 2007; Lee *et al*., 2008). As a result, EspI contributes to the disruption of cellular tight junctions (Thanabalasuriar *et al*., 2010). Map is targeted to the mitochondria via an N-terminal mitochondrial targeting sequence where it disrupts the mitochondrialmembrane potential (Kenny and Jepson, 2000; Papatheodorou *et al*., 2006). At early time points Map induces formation of transient filopodia at the bacterial attachment sites (Kenny and Jepson, 2000) via its guanine exchange factor (GEF) activity for the Rho-GTPase Cdc42 (Huang *et al*., 2009). Moreover, via its C-terminus, which comprises a class I PSD-95/Disc Large/ZO-1 (PDZ)-binding motif (TRL), Map binds PDZ 1 of Na^+^/H^+^ exchanger regulatory factor 1 (NHERF1) (Alto *et al*., 2006; Simpson *et al*., 2006). PDZ domains are protein–protein recognition domains of about 90 amino acids, which are widely represented in the human genome (Tonikian *et al*., 2008). PDZ-binding motifs comprise a consensus S/T-X-Φ sequence (where X is any amino acid and Φ is any hydrophobic residues (Songyang *et al*., 1997). Importantly, PDZ binding motifs are also found at the C-termini of NleH1 and EspI (SKI and TRV respectively). Whereas no mammalian partner was thus far associated with the PDZ-binding motif of NleH1, a previous report demonstrated that EspI binds a number of PDZ-containing proteins, including NHERF1 and NHERF2 (Lee *et al*., 2008).

NHERF proteins (NHERF1 to 4) are present in abundance in the mammalian small intestine and colon (Donowitz *et al*., 2005) where they have a central role in trafficking, membrane retention, dimerization and regulation of ion channels and membrane proteins (Shenolikar and Weinman, 2001; Shenolikar *et al*., 2004). NHERF1 and 2 are closely related (51% amino acid identity) and the only members in the NHERF protein family which possess two PDZ domains and a C-terminal ezrin/radixin/moesin (ERM) binding domain (EBD) (Reczek *et al*., 1997; Yun *et al*., 1998). The EBD domain links NHERF1 and 2 to the actin cytoskeleton thereby forming a scaffold for highly organized multimeric signalling complexes (reviewed in Lamprecht and Seidler, 2006). More than 30 binding partners have been reported for NHERF1 and 2 (Shenolikar *et al*., 2004), some are shared by more than one NHERF isoform (Yun *et al*., 1998; Sun *et al*., 2000), while others are NHERF isoform-specific (Shenolikar *et al*., 2004).

In this paper we report the binding of three T3SS effector proteins (Map, EspI and NleH1) to a single eukaryotic target (NHERF2). We show that NHERF2 influences the trafficking and function of EspI, Map and NleH1, suggesting that it functions as a distribution hub at the plasma membrane.

## Results

### Map, EspI and NleH1 bind NHERF2

**Fig. 1:**
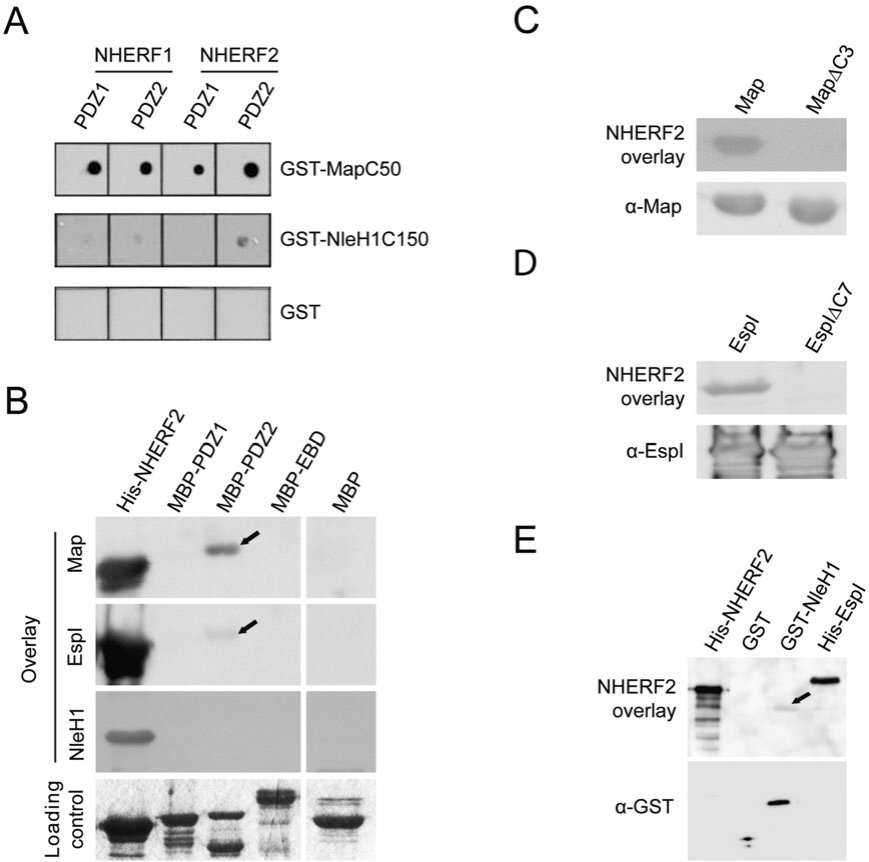
A. PDZ-domain protein arrays were overlaid with purified GST-MapC50, GST-NleH1C150 or GST followed by detection with anti-GST antibodies. MapC50 interacts with PDZ1 and 2 domains of NHERF1 and NHERF2 whereas NleH1C150 interacts only with the PDZ2 domain of NHERF2.B. Purified His-NHERF2, MBP-PDZ1, MBP-PDZ2, MBP-EBD and MBP were transferred onto PVDF membrane and overlaid with purified MBP-Map followed by detection with anti-Map, His-EspI followed by detection with anti-EspI or GST-NleH1 followed by detection with anti-NleH. Equivalent protein loading was confirmed by Coomassie staining. Map, EspI and NleH1 interact with His-NHERF2, but only Map and EspI interacted with MBP-PDZ2 (black arrows).C and D. Bacterial extracts of EPEC Δ*map* overexpressing Map and MapΔC3 (C) or EPEC Δ*espI* overexpressing EspI and EspIΔC7 (D) were transferred onto PVDF membrane and overlaid with purified His-NHERF2 followed by detection with anti-NHERF2 or immunoblotted with an anti-Map or anti-EspI antibody. NHERF2 was able to interact with Map and EspI but not MapΔC3 or EspIΔC7.E. Purified His-NHERF2, GST, GST-NleH1 and His-EspI were transferred onto PVDF membrane and overlaid with purified His-NHERF2 followed by detection with anti-NHERF2 or immunoblotted with anti-GST. NHERF2 interacted with His-EspI and GST-NleH1 (black arrow).

Previous reports have demonstrated that Map interacts with NHERF1 (Alto *et al*., 2006; Simpson *et al*., 2006), and a global PDZ array screen indicated that EspI might bind NHERF1 and NHERF2 (Lee *et al*., 2008). In this study we screened target proteins, which are recognized by the PDZ-motifs of Map and NleH1 using the PDZ array. Overlaying a fusion consisting of the last 50 amino acids of Map and GST (GST-MapC50) on the PDZ-domain array confirmed that Map binds PDZ1 of NHERF1 (Fig. 1A). In addition, we found that Map interacted with PDZ2 of NHERF1 and both PDZ domains of NHERF2 (Fig. 1A). NHERF2 was also identified as interaction partner of Map by a yeast two-hybrid screen using full-length Map as bait and HeLa cDNA library as a prey (data not shown). Overlaying a fusion consisting of the last 150 amino acids of NleH1 and GST (GST-NleH1C150) (a GST-NleH1C50 fusion was unstable) on the PDZ-domain protein array revealed that NleH1 bound PDZ2 of NHERF2, although this interaction appeared weaker than either Map:NHERF2 or EspI:NHERF2 (Fig. 1A) (Lee *et al*., 2008).

In order to confirm and characterize the interaction of Map, EspI and NleH1 with NHERF2, we constructed and purified His-NHERF2 and MBP fusions of NHERF2 PDZ1, PDZ2 and EBD, which were immobilized on a membrane and overlaid with purified MBP-Map, His-EspI and GST-NleH1. Detection of bound proteins with effector-specific antiserum revealed that Map, EspI and NleH1 interacted with full-length NHERF2 (Fig. 1B). Map and EspI, but not NleH1, also bound the purified PDZ2 domain of NHERF2 (Fig. 1B). Although Map interacted with PDZ1 of NHERF2 on the PDZ array, neither Map nor EspI bound the purified PDZ1 or EBD domains (Fig. 1B). These results show that despite some differences between binding to the arrayed and fusion proteins, NHERF2 is targeted by Map, EspI and NleH1.

In a reciprocal experiment full-length Map, EspI and NleH1 were overlaid with His-NHERF2, which confirmed the interactions (Fig. 1C, D and E). This binding was dependent on the PDZ-binding motifs of Map and EspI as NHERF2 did not bind the C-terminally truncated effectors MapΔC3 (Fig. 1C) or EspIΔC7 (Fig. 1D). Unfortunately, due to protein instability of NleH1ΔC4, the role of the PDZ binding motif of recombinant NleH1 in binding NHERF2 could not be investigated.

### NHERF2 co-immunoprecipitates EspI and NleH1 and colocalizes with Map, EspI and NleH1

**Fig. 2:**
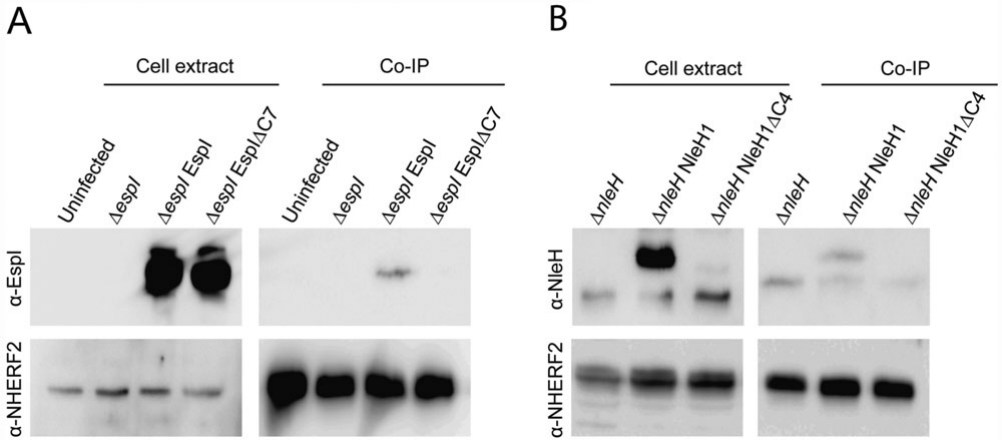
A. HA-NHERF2 was immunoprecipitated with anti-HA from HeLa–NHERF2 cells infected for 1 h with the indicated EPEC strains. Equivalent protein loading was confirmed by anti-NHERF2 Western blots. Anti-EspI Western blot reveals equivalent levels of EspI and EspIΔC7 in the whole-cell extract and the presence of EspI, but not EspIΔC7, in HA-NHERF2 immunoprecipitated sample.B. HA-NHERF2 was immunoprecipitated with anti-HA from HeLa–NHERF2 cells infected for 1 h with the indicated EPEC strains. Equivalent loading of proteins was monitored by anti-NHERF2 Western blots. Anti-NleH1 Western blot reveals presence of NleH1 in whole-cell extracts (left panel), which was immunoprecipitated with HA-NHERF2 sample (right panel).

Once we confirmed the interaction between NHERF2 and Map, EspI and NleH1 using recombinant proteins, we aimed to confirm the interaction by coimmunoprecipitation. Towards this end we generated a stable HeLa cell line constitutively expressing HA-tagged NHERF2 (HeLa–NHERF2). Immunofluorescence microscopy using anti-HA antibodies revealed that NHERF2 localized mainly at the plasma membrane (data not shown), in a pattern similar to that reported in un-polarized epithelial cells like A431 or HT1080 cells, which express NHERF2 endogenously (Theisen *et al*., 2007). The HeLa–NHERF2 cells were infected for 1 h with EPEC Δ*map* and EPEC Δ*map* expressing full-length Map or MapΔC3; EPEC Δ*espI* and EPEC Δ*espI* expressing full-length EspI or EspIΔC7; and EPEC Δ*nleH* and EPEC Δ*nleH* expressing full-length NleH1 or NleH1ΔC4. We were unable to co-immunoprecipitate NHERF2 and full-length Map or MapΔC3 (data not shown). Probing infected cell extracts with anti-EspI and anti NleH1 revealed that while both full-length EspI and EspIΔC7 were found in equal amounts, NleH1ΔC4 was unstable (Fig. 2A and B). Following cell lysis the supernatants were subjected to co-immunoprecipitation with anti-HA antibodies. While NHERF2 did not co-immunoprecipitate EspIΔC7 (Fig. 2A) and NleH1ΔC4 (Fig. 2B), both full-length EspI (Fig. 2A) and NleH1 (Fig. 2B) were co-immunoprecipitated.

**Fig. 3:**
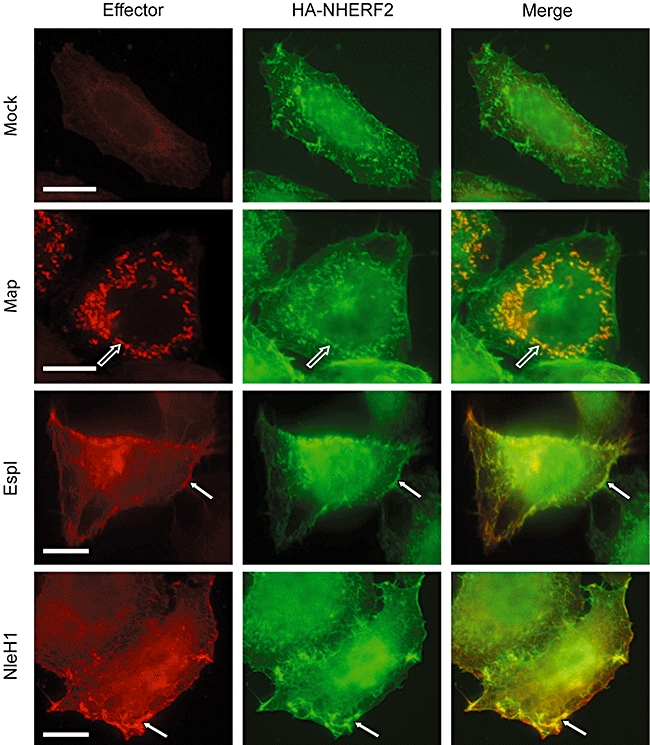
Fluorescence microscopy of HeLa–NHERF2 cells transfected with pRK5 (Mock), pRK5-*map* (Map), pEGFP-C2-*espI* (EspI) and pRK5-*nleH1* (NleH1). NHERF2 was detected with anti-HA (green) and effectors Map and NleH1 were stained with anti-myc (red). Green GFP signal was converted to red for EspI. Map colocalizes with NHERF2 (open arrows), while partial colocalizations of EspI and NleH1 with NHERF2 are observed at the plasma membrane (plain arrows). Scale bar = 10 µm.

In order to determine if the effectors colocalize with NHERF2, HeLa–NHERF2 cells were transfected with pRK5 (Fig. 3) or pRK5 expressing myc-tagged Map, MapΔC3, NleH1 and NleH1ΔC4 as well as pGFP-EspI and pGFP-EspIΔC7. Analysing the transfected cells by immunofluorescence revealed that Map colocalized with mitochondria (Fig. S1) and NHERF2 (Fig. 3). Plasma membrane-associated EspI and NleH1 colocalized with NHERF2 (Fig. 3). We observed no colocalization of NHERF2 and MapΔC3, EspIΔC7 or NleH1ΔC4 (Fig. S2).

### NHERF2 modulates Map-induced filopodia dynamics

**Fig. 4:**
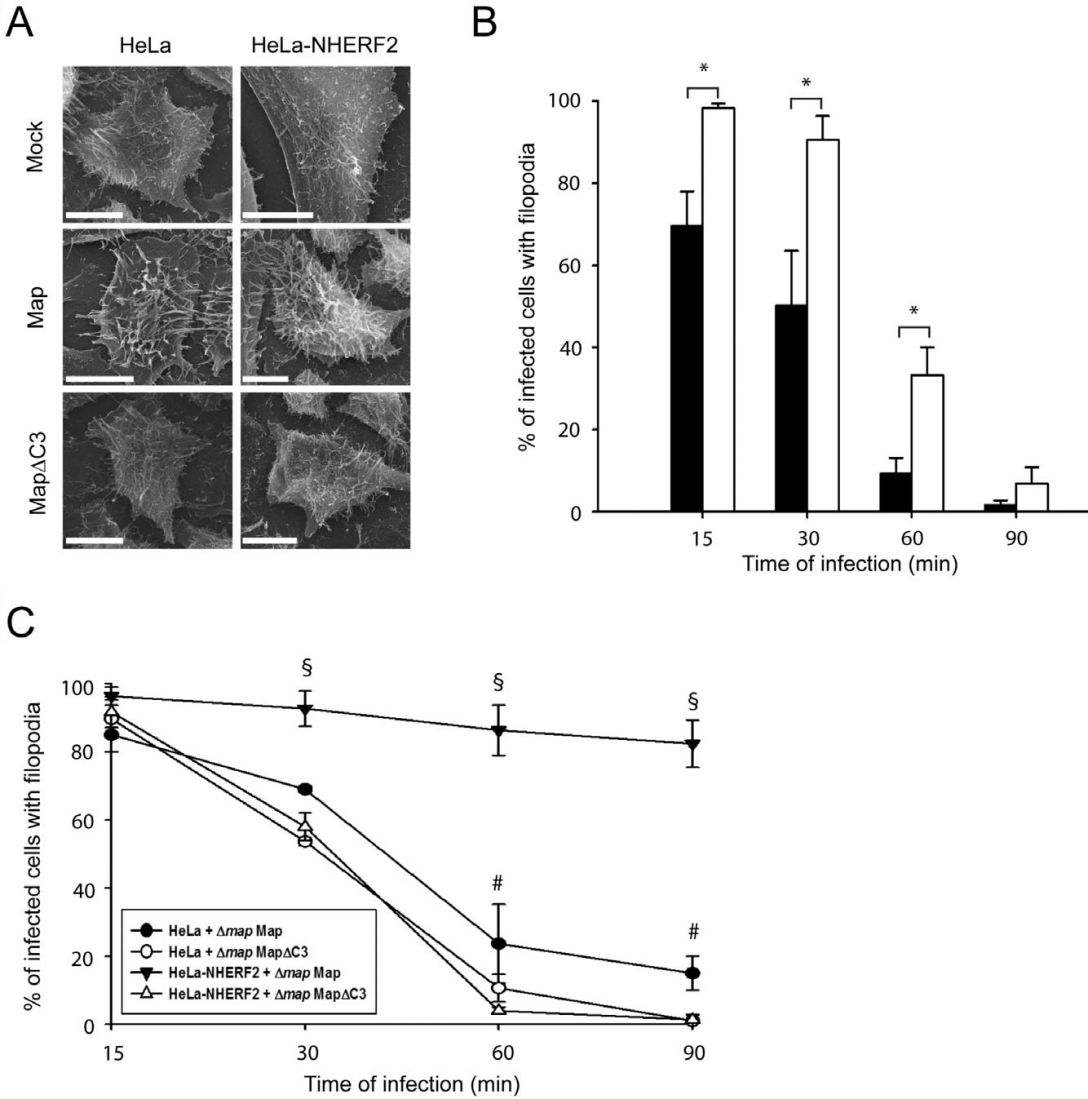
A. Scanning electron microscopy of Hela and HeLa–NHERF2 cells transfected with pRK5 (Mock), pRK5-*map* or pRK5-*map*ΔC3. Transfection of cells with pRK5-*map* induced filopodia in both cell lines; however, filopodia formed on HeLa–NHERF2 were longer and more complex. Only sparse filopodia formation was observed upon transfection with pRK5-*map*ΔC3. Scale bar = 10 µm.B. Quantification of infected HeLa (black bars) and HeLa–NHERF2 (white bars) cells displaying filopodia following infection with wild-type EPEC. Downregulation of filopodia is delayed in HeLa cells expressing HA-NHERF2. One hundred infected cells were counted in three independent experiments. Results are shown as mean ± SD. *P*-values of < 0.05 (*) were considered as significant.C. Quantification of infected HeLa and HeLa–NHERF2 cells displaying filopodia following infection with EPEC Δ*map* overexpressing Map or MapΔC3. EPEC Δ*map* overexpressing Map, but not MapΔC3, maintains filopodia over time in HeLa–NHERF2 cells compared with HeLa cells. One hundred infected cells were counted in three independent experiments. Results are shown as mean ± SD. *P*-values of < 0.05 (# for Map versus MapΔC3 in HeLa cells, § for Map versus MapΔC3 in HeLa–NHERF2 cells) were considered as significant.

We investigated the effect of NHERF2 expression on Map-induced filopodia. First, HeLa and HeLa–NHERF2 were transfected with pRK5 expressing myc-tagged Map or MapΔC3 and examined by scanning electron microscopy. This revealed that ectopic expression of Map resulted in filopodia formation in both cell lines, although the length and complexity of filopodia were more prominent on the HeLa–NHERF2 cells (Fig. 4A). Relatively small and sporadic filopodia were observed in either cell line ectopically expressing MapΔC3 (Fig. 4A). Next HeLa and HeLa–NHERF2 cells were infected with EPEC wild-type and the dynamics of filopodia formation and withdrawal was quantified by counting infected cells with filopodia over time. This revealed that at each time point more HeLa–NHERF2 cells with filopodia were observed compared with HeLa cells (Fig. 4B).

To investigate whether persistence of filopodia was due to the PDZ-mediated Map–NHERF2 interaction, the number of cells with filopodia was quantified following infection of HeLa and HeLa–NHERF2 cells with EPEC Δ*map* expressing Map or MapΔC3. Upon infection with EPEC Δ*map* expressing full-length Map, the fraction of infected HeLa–NHERF2 cells with filopodia was higher and decreased much slower compared with infected HeLa cells (Fig. 4C). This is consistent with the phenotype observed for infection with wild-type EPEC (Fig. 4B). In contrast, no difference in numbers of cells with filopodia or the kinetics of filopodia withdrawal was found between HeLa–NHERF2 and HeLa cells infected with EPEC Δ*map* expressing MapΔC3 (Fig. 4C). These results suggest that the expression of NHERF2 in HeLa cells modulates Map-induced filopodia formation and persistence, which is dependent on the PDZ-mediated Map–NHERF2 interaction.

### NHERF2 accelerates trafficking of EspI to the Golgi

**Fig. 5:**
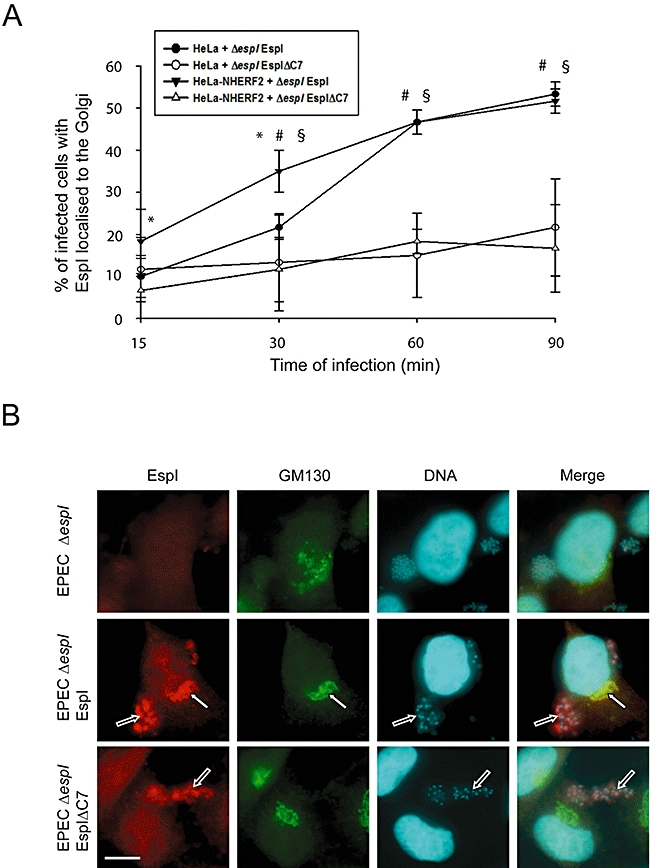
A. Quantification of EspI Golgi localization in HeLa and HeLa–NHERF2 cells infected with EPEC Δ*espI* expressing EspI or EspIΔC7. Increased EspI localization at the Golgi apparatus was observed in HeLa–NHERF2 cells compared with HeLa cells following 15 and 30 min of infection (*). EspIΔC7 shows reduced localization to the Golgi compared with EspI in both cell lines (# for HeLa cells, § for HeLa–NHERF2 cells). Fifty infected cells were counted in three independent experiments. Results are shown as mean ± SD. *P*-values of < 0.05 (* for HeLa versus HeLa–NHERF2 in EPEC Δ*espI* overexpressing EspI infected cells, # for EspI versus EspIΔC7 in HeLa cells and § for EspI versus EspIΔC7 in HeLa–NHERF2 cells) were considered as significant.B. Fluorescence microscopy of HeLa–NHERF2 infected for 30 min with EPEC Δ*espI* and EPEC Δ*espI* expressing EspI or EspIΔC7. EspI was stained with anti-EspI (red), Golgi apparatus was stained with anti-GM130 (green) and DNA was counterstained with Hoechst 33342 (cyan). EspI is detected at the bacterial attachment sites (open arrows); however, Reduced colocalization with the Golgi was observed for cells infected with EPEC Δ*espI* expressing EspIΔC7 (plain arrows). Scale bar = 10 µm.

During the early stages of EPEC infection, localization of EspI shifts from the site of bacterial adhesion to the Golgi apparatus, and this trafficking is enhanced by its PDZ binding motif (Lee *et al*., 2008). In order to investigate the possible role of NHERF2 in trafficking of EspI to the Golgi, HeLa and HeLa–NHERF2 cells were infected for 15, 30, 60 and 90 min with EPEC Δ*espI* expressing EspI. The number of infected cells with EspI colocalizing with the Golgi protein GM130 was quantified by immunofluorescence microscopy (Fig. 5A). At 15 and 30 min post infection, significantly more HeLa–NHERF2 cells showed EspI staining at the Golgi apparatus compared with HeLa cells (Fig. 5A). To determine the importance of the EspI–NHERF2 interaction for trafficking of the protein to the Golgi apparatus, HeLa–NHERF2 cells were infected for 15, 30, 60 and 90 min with EPEC Δ*espI* expressing EspI or EspIΔC7 (Fig. 5A). Golgi staining of EspIΔC7 was significantly lower after 30 (Fig. 5A and B), 60 and 90 min post infection compared with cells infected with EPEC Δ*espI* expressing EspI (Fig. 5A).

Taken together, these results suggest that following translocation EspI interacts with NHERF2 via its PDZ-binding motif, which accelerates trafficking of EspI to the Golgi apparatus.

### NHERF2 antagonises the anti-apoptotic activity of NleH1

**Fig. 6:**
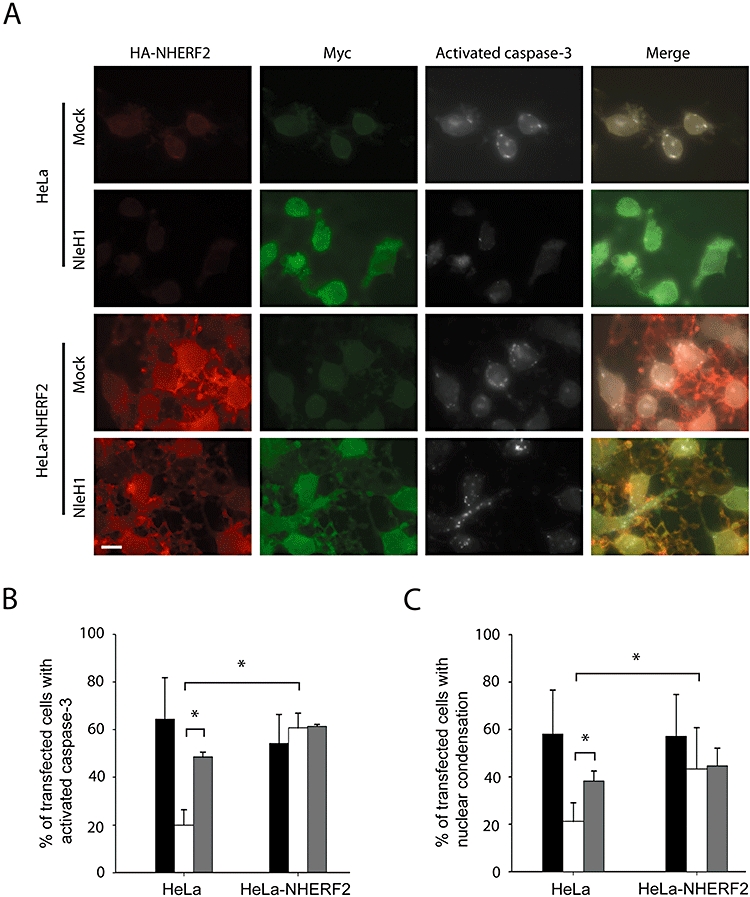
A. Fluorescence microscopy of HeLa and HeLa–NHERF2 cells transfected with pRK5 or pRK5-*nleH1* and treated for 4 h with 1 µM STS. HA-NHERF2 was stained with anti-HA (red), NleH1 with anti-myc (green) and activated caspase-3 was detected with anti-cleaved caspase-3 (white). Expression of NleH1 in HeLa cells inhibited activation of caspase-3 whereas expression of NleH1 in HeLa–NHERF2 cells did not. Scale bar = 10 µm.B and C. HeLa and HeLa–NHERF2 cells were transfected with pRK5 (black bars), pRK5-*nleH1* (white bars) or pRK5-*nleH1*ΔC4 (grey bars) before treated with 1 µM STS for 4 h. Capsase-3 activation and nuclear condensation in transfected cells were observed by immunofluorescence microscopy using anti-myc, anti-cleaved caspase-3 (B) and Hoechst 33342 DNA staining (C). Immunofluorescence analysis revealed that cells expressing HA-NHERF2 and NleH1 were more often positive for caspase-3 activation and nuclear condensation than HeLa cells expressing NleH1. One hundred transfected cells were counted for activated caspase-3 or nuclear condensation in three independent experiments. Results are shown as mean ± SD. *P*-values of < 0.05 (*) were considered as significant.

NleH1 interacts with Bax inhibitor-1, which protects the cell against various apoptotic stimuli (Hemrajani *et al*., 2010; Robinson *et al*., 2010). We investigated the impact of NHERF2 expression on the anti-apoptotic activity of NleH1. Low translocation levels of NleH1ΔC4 prevented an analysis of the importance of the PDZ-binding motif during infection. To circumvent this, we ectopically expressed NleH1 and NleH1ΔC4 in HeLa or HeLa NHERF2 cells, which were treated with the pro-apoptotic compound staurosporine (STS). The number of transfected apoptotic cells, marked by caspase-3 activation or nuclear condensation, was quantified by immunofluorescence counting. Significantly fewer HeLa cells transfected with pRK5-NleH1 displayed caspase-3 activation (Fig. 6A and B) or nuclear condensation (Fig. 6C) compared with pRK5-transfected HeLa cells or cells transfected with pRK5-NleH1ΔC4. Mock-transfected HeLa–NHERF2 cells displayed a similar phenotype as normal HeLa cells, showing that NHERF2 expression *per se* neither promotes nor prevents STS-induced apoptosis (Fig. 6B and C). Expression of NHERF2 did not influence the phenotype of cells expressing NleH1ΔC4. However, the inhibition of caspase-3 cleavage (Fig. 6A and B) and nuclear condensation (Fig. 6C) by NleH1 was significantly abolished in cells expressing HA-NHERF2 compared with the HeLa cell control. Taken together, these results show that even though the anti-apoptotic activity of NleH1 depends on its PDZ-binding motif, the interaction with NHERF2 is not the trigger for its anti-apoptotic signalling. The data rather suggest that NHERF2 counteracts the anti-apoptotic activity of NleH1, possibly by direct competition for its PDZ binding site or interference with its intracellular trafficking and localization.

## Discussion

NHERF proteins are known to orchestrate intracellular protein trafficking, protein localization and cell signalling; regulation of ion transporters being the best-studied example. NHERFs, which are modular proteins comprising several protein–protein interaction domains, function as scaffolds for the assembly of multiprotein complexes at the plasma membrane. In particular, NHERFs are involved in tethering plasma membrane localized transporters such as NHE3, the apical form of the Na^+^/H^+^ exchanger and CFTR, a cAMP-regulated Cl-channel, to the underlying actin cytoskeleton. NHERF1 and 2 bind the transporters via their PDZ domains and actin-associated ERM proteins via their C-terminal EBD (Brone and Eggermont, 2005), thus linking the actin cytoskeleton to the plasma membrane (Bretscher *et al*., 2000). The assignment of individual functions to NHERF1 or 2 has proven to be difficult due to high homology and widely overlapping interactomes. Localization studies have shown that, in polarized Caco-2 cells, NHERF1 is predominantly found in the brush border (BB) whereas NHERF2 has some BB localization but is mainly found in the inter-microvillar clefts below the BB (Donowitz *et al*., 2005). The differential subcellular localization and tissue distribution of NHERFs might determine functional specificity and account for the observation from studies with knockout mice that NHERF1–3 are not functionally redundant. (Seidler *et al*., 2009). Despite their central position in diverse signalling processes, the role of NHERF proteins as versatile targets for bacterial effector proteins has not been fully investigated.

Although fulfilling unrelated intracellular functions Map, EspI and NleH1 share two common post-translocation features, binding to NHERF2 and trafficking to an intracellular membranous compartment. Map is targeted to the mitochondria (Kenny and Jepson, 2000), EspI to the Golgi (Gruenheid *et al*., 2004) and NleH1 to the endoplasmic reticulum (ER) (Hemrajani *et al*., 2010). A PDZ array screen indicated that Map, EspI and NleH1 bind NHERF2. Using recombinant Map, EspI and NleH1 and a stable NHERF2 cell line revealed that all three effectors interact, in a PDZ binding motif-dependent manner, with NHERF2. Moreover, all three effectors colocalized with NHERF2 in HeLa–NHERF2 cells, and EspI and NleH1 were co-immunoprecipitated with NHERF2 from infected cells. Our efforts to co-immunoprecipitate Map and NHERF2 were not successful. The fact that expression of NHERF2 in HeLa cells modulated the intracellular trafficking and/or activity of all three effectors suggests that NHERF2 might form a molecular hub at the plasma membrane from which the effectors are distributed to their final destinations.

Map acts as a Rho-guanine nucleotide exchange factor (GEF) for Cdc42 (Huang *et al*., 2009), but is unique in the family of WxxxE motif proteins in possessing a PDZ-binding motif at the C-terminus (Alto *et al*., 2006; Bulgin *et al*., 2010). Approximately 37% of human and mouse GEFs also possess a PDZ binding motif and there is increasing evidence that PDZ-based interactions are important for GEF function, and aid targeting of the GEF to a subcellular location (Garcia-Mata and Burridge, 2007). Here removal of the C-terminal PDZ motif abolished the interaction of Map with NHERF2 and led to reduced filopodia formation and stability. Endogenous GTPases are modified posttranscriptionally by the addition of a lipid moiety to the C-terminus (farnesyl, geranyl, palmitoyl or methyl) (Roberts *et al*., 2008), which targets them to different membranous compartments. We suggest that the interaction of Map with the PDZ2 domain of NHERF2 may transiently anchor Map at the apical surface of an infected cell leading to filopodia formation beneath adherent bacteria, before the effector is targeted to the mitochondria.

EspI has been reported to inhibit COPII-dependent vesicular transport between the ER and Golgi leading to alterations in host cell protein secretion and tight junction integrity (Kim *et al*., 2007; Thanabalasuriar *et al*., 2010). EspI dynamics and trafficking upon translocation are still poorly understood since the protein accumulates at the Golgi apparatus and not at the ER, where COPII vesicles are formed (Kim *et al*., 2007). Here we showed that the interaction between EspI and NHERF2 is mediated through the EspI C-terminal PDZ-binding motif, resulting in an increased rate of trafficking of EspI to the Golgi. This result is consistent with the potential role of NHERF2 in recycling of endogenous membrane proteins (Donowitz *et al*., 2005).

Recently, we found that NleH1 inhibits cell apoptosis by interacting with Bax inhibitor-1 and decreasing activation of caspase-3, nuclear condensation and mitochondrial hyper permeabilization (Hemrajani *et al*., 2010; Robinson *et al*., 2010). NleH1 also binds to ribosomal protein 3 and reduces nuclear activity of NF-κB (Gao *et al*., 2009). Similar to Map and EspI, we found that NleH1 possesses a class I PDZ binding motif that regulates its anti-apoptotic function. Although a recombinant PDZ-binding motif mutant form of NleH1 could not be purified because of protein instability, immunofluorescence of cells transfected with the PDZ motif mutant demonstrated that the motif was critical to wild-type function of NleH1 and its interaction with NHERF2. However, the interaction with NHERF2 is not the trigger for NleH1-mediated anti-apoptotic signalling; on the contrary, binding of NleH1 to NHERF2 inhibits anti-apoptotic activity. This might represent a mechanism for the temporal control of NleH1 anti-apoptotic function. In addition, NleH1-NHERF2 interaction might enable NleH1 in general, or a fraction of the translocated protein, to perform a secondary, yet unidentified, activity before or in parallel to promoting cell survival.

The fact that three different T3SS effectors, which share little sequence similarity and have distinct functions, bind a single host cell scaffold protein is unprecedented. Moreover, while NleH1 binds NHERF2 only, EspI and Map bind both NHERF1 and NHERF2. It is tempting to speculate that EPEC and EHEC exploit endogenous BB-associated NHERFs to regulate spatial and temporal intracellular trafficking of Map, EspI and NleH1. Indeed, during infection of mice with wild-type *C. rodentium* NHERF2 was extensively recruited to the bacterial attachment sites (data not shown).

T3SS effectors function in a co-ordinated manner. As effectors from a single pathogen can complement or antagonize each other, the timing and location of their activity must be highly regulated. While timing of gene expression and effector protein translocation provides a regulatory mechanism at the bacterial level (Mills *et al*., 2008), ubiquitination followed by proteasomal degradation (Kubori and Galan, 2003) and protein phosphorylation (Kenny, 1999) represent mechanisms, which are exploited to control the activity of translocated effectors within host cells. In this study we identified a novel regulatory mechanism by which recruitment of NHERFs to the bacterial attachment site forms a distribution hub, which is exploited for membrane retention (Map) or trafficking (EspI). We propose that differential binding to NHERF1 and/or NHERF2 and to their different PDZ domains provides a mechanism to fine tune the activity of the hub. We aim to exploit the *C. rodentium* model to test this hypothesis in the context of host pathogen interactions *in vivo*.

## Experimental procedures

### Bacterial strains and growth conditions

*Escherichia coli* strains used in this study are listed in Table 1. Bacteria were cultured in Luria-Bertani (LB) medium or in Dulbecco's modified Eagle's medium (DMEM) supplemented with ampicillin (100 µg ml^−1^) or kanamycin (50 µg ml^−1^) as appropriate.

**Table 1:** List of strains.

Strain	Description	Origin
E2348/69	Wild-type EPEC O127:H6	Levine *et al*. (1978)
ICC202	E2348/69 Δ*map*	Simpson *et al*. (2006)
ICC248	E2348/69 Δ*espI*	Marches *et al*. (2008)
ICC303	E2348/69 Δ*nleH1*Δ*nleH2* (named Δ*nleH*)	Hemrajani *et al*. (2010)

### Plasmids

Plasmids and primers used in this study are listed in Tables 2 and 3. For cloning into the bacterial expression vector pSA10 (Schlosser-Silverman *et al*., 2000), *espI, espIΔC7* and *nleH1ΔC4* were amplified from EPEC genomic DNA using primer pairs EspI-Fw1 and EspI-Rv1, EspIΔC7-Rv1 and NleH1-Fw1 and NleH1ΔC4-Rv, respectively. For generation of (His)_6_-tagged proteins, *nherf2* was amplified from pBKCMV-HA-NHERF2 using primers NHERF2-Fw and NHERF2-Rv and *espI* was amplified from EPEC genomic DNA using primers EspI-Fw2 and EspI-Rv2; both PCR products were cloned into pET28a(+) (Novagen). For generation of MBP fusion proteins, *map* was amplified from pICC330 using primer pair Map-Fw2 and Map-Rv2, and PDZ1, PDZ2 and the ezrin-binding domain (EBD) of NHERF2 were PCR-amplified from pBKCMV-HA-NHERF2 using primer pairs PDZ1-Fw and PDZ1-Rv, PDZ2-Fw and PDZ2-Rv, EBD-Fw and EBD-Rv; all constructs were cloned into pMalc2X (New England Biolabs). For generation of GST fusion proteins, *nleH1* and *nleH1C150* (encoding the 150 C-terminal amino acids of NleH1) were amplified from EPEC genomic DNA using primer pairs NleH1-Fw2 or NleH1C150-Fw and NleH1-Rv2 respectively. Both constructs were cloned into pGEX-KG. For *mapC50,* the region encoding the 50 C-terminal amino acids of Map was amplified from EPEC genomic DNA using primer pairs MapC50-Fw or MapC50-Rv. The resulting fragment was cloned into the SmaI/EcoRI sites of pGEX-3X. For transfection experiments, *map*, *nleH1* and *nleH1ΔC4* were amplified from EPEC genomic DNA using primer pairs Map-Fw1 and -Rv1, NleH1-Fw1 and NleH1-Rv1 or NleH1ΔC4-Rv, respectively, and cloned into pRK5. Plasmid pRK5-MapΔC3 was generated by inverse PCR using pRK5-Map as template. *espI* and *espI*ΔC7 were amplified from EPEC genomic DNA using primer pairs EspI-Fw3 and EspI-Rv3 or EspIΔC7-Rv3, respectively, and cloned into pGFP-C2. All constructs were verified by DNA sequencing.

**Table 2:** List of plasmids.

	Description	Origin
pBKCMV	Mammalian expression vector	Stratagene
pBKCMV-HA-NHERF2	pBKCMV encoding HA-tagged NHERF2	Dr R. Hall
pSA10	pKK177-3 containing *lacI*	Schlosser-Silverman *et al*. (2000)
pRK5	N-terminal Myc-tag mammalian expression vector	Clontech
pICC330	pSA10-*map*	Simpson *et al*. (2006)
pICC331	pSA10-*mapΔC3*	Simpson *et al*. (2006)
pICC507	pSA10-*espI*	This study
pICC508	pSA10-*espI*ΔC7	This study
pICC443	pSA10-*nleH1*	Hemrajani *et al*. (2010)
pICC535	pSA10-*nleH1ΔC4*	This study
pICC509	pRK5-*map*, encoding myc-tagged Map	This study
pICC510	pRK5-*map*ΔC3, encoding myc-tagged MapΔC3	This study
pICC512	pRK5-*nleH1*, encoding myc-tagged NleH1	This study
pICC513	pRK5-*nleH1*ΔC4, encoding myc-tagged NleH1ΔC4	This study
pEGFP-C2	Vector expressing fusions to the C-terminus of GFP	Clontech
pGFP-EspI	pEGFP-C2-*espI* encoding EspI	This study
pGFP-EspI ΔC7	pEGFP-C2-*espI*ΔC7 encoding EspIΔC7	This study
pET28a	His6-tag expression vector	Novagen
pICC332	pET28a-*nherf1*, encoding His6-NHERF1	Simpson *et al*. (2006)
pICC514	pET28a-*nherf2*, encoding His6-NHERF2	This study
pICC515	pET28a-*espI*, encoding His6-EspI	This study
pMalc2X	MBP-tag expression vector	New England Biolabs
pICC516	pMalc2X-*map*, encoding MBP-Map	This study
pICC517	pMalc2X-PDZ1, encoding MBP-PDZ1 of NHERF2	This study
pICC518	pMalc2X-PDZ2, encoding MBP-PDZ2 of NHERF2	This study
pICC519	pMalc2X-EBD, encoding MBP-EBD of NHERF2	This study
pGEX-KG	GST-tag expression vector	GE Healthcare
pGEX-3X	GST-tag expression vector	GE Healthcare
pICC520	pGEX-KG-*nleH1*, encoding GST-NleH1	This study
pICC521	pGEX-KG-*nleH1*C150, encoding GST-NleH1c150	This study
pGST-Map50	pGEX-3X-*map*C50, encoding GST-MapC50	This study

**Table 3:** List of primers (restriction sites in bold).

Name	Sequence
NHERF2-Fw	5′-CTTAAA**GCTAGC**ATGGCCGCGCCGGAG-3′
NHERF2-Rv	5′-GGGTTT**GAATTC**TCAGAAGTTGCTGAAG-3′
PDZ1-Fw	5′-CCG**GAATTC**CTGTGCCGCTTGGTGCGCG-3′
PDZ1-Rv	5′-ATTT**CTGCAG**TCACTGGTCCACCACCAGCAGCCGAG-3′
PDZ2-Fw	5′-CCG**GAATTC**CGGCTCTGCCACCTGCGAAAG-3′
PDZ2-Rv	5′-ATTT**CTGCAG**TCAGGGGTCCACGACCAGCAGCCGGG-3′
EBD-Fw	5′-CG**GAATTC**GAGACAGATGAACACTTCAAGCGGCTTC-3′
EBD-Rv	5′-GTTAAA**CTGCAG**TCAGAAGTTGCTGAAGATTTC-3′
Map-Fw1	5′-TTT**GAATTC**GGAGCAAATGTTTAGTCCAACGGCAAT -3′
Map-Fw2	5′-TTT**GAATTC**GGAGCAATGTTTAGTCCAACGGCAAT -3′
Map-Rv	5′-CCAATGCATTGGTT**CTGCAG**CTACAGCCGAGTATCCTGCA-3′
MapΔC3-Fw	5′-TAGCTGCAGAAGCTTGGCCG-3′
MapΔC3-Rv	5′-ATCCTGCACATTGTCTGCAATC-3′
MapC50-Fw	5′-**GGATCC**CCGATCCCATTACACGTTTTAAC-3′
MapC50-Rv	5′-**GAATTC**CTACAGCCGAGTATCCTGCAC-3′
EspI-Fw1	5′-G**GAATTC**ATGAACATTCAACCGATCGTAACATCCGG-3′
EspI-Fw2	5′-GG**GGATCC**AACATTCAACCGATCGTAACATCC-3′
EspI-Fw3	5′-CGGAATTCATGAACATTCAACCGATCG-3′
EspI-Rv1	5′-AGGG**CCCGGG**TTAGACTCTTGTTTCTTGGATTATATCAACGG-3′
EspI-Rv2	5′-CG**GAATTC**TTAGACTCTTGTTTCTTGGATTATATC-3′
EspI-Rv3	5′-CGGGATCCTTAGACTCTTGTTTCTTGG-3′
EspIΔC7-Rv1	5′-AGGG**CCCGGG**TTAATCAACGGTATCAACATAATTTGATGG-3′
EspIΔC7-Rv3	5′-CGGGATCCTTAATCAACGGTATCAACATAATTTGATGG-3′
NleH1-Fw1	5′-GC**GGATCC**GCGCTATCACCATCTTCTGTAAATTTG-3′
NleH1-Rv1	5′-GCG**AAGCTT**CTAAATTTTACTTAATACCACAC-3′
NleH1ΔC4-Rv	5′**-CTGCAG**TTTCGACTATACCACACTAATAAGATCTTG-3′
NleH1-Fw2	5′-GACT**CCATGG**GTCTATCACCATCTTCTGTAAATTTG-3′
NleH1c150-Fw	5′-GACT**CCATGG**GTTATGTTATTGGCAAAGGGGGTA-3′
NleH1-Rv2	5′-GCG**AAGCTT**CTAAATTTTACTTAATACCACAC-3′

### Protein expression and purification

Genes cloned into pET28a, pMALc2X and pGEX vectors were expressed in *E. coli* BL-21/DE3 star strain (Invitrogen) in presence of 1 mM isopropyl-β-d-thiogalactopyranoside (IPTG) at 37°C as described previously (Crepin *et al*., 2005). His-tagged proteins were purified using Ni^2+^ agarose His-Bind Resin Column (Novagen), MBP-tagged proteins were purified on Amylose resin (New England Biolabs) and GST-tagged proteins were purified on glutathione-sepharose (GE Healthcare) using manufacturer's recommendations.

### PDZ array screen

To assess the binding of the GST-MapC50 and NleH1C150 fusion proteins to the PDZ domain array, purified His-tagged PDZ domain fusion proteins were spotted as previously described (Fam *et al*., 2005; He *et al*., 2006) at 1 µg per bin onto Nytran SuperCharge 96-grid nylon membranes (Schleicher & Schuell). The membranes were allowed to dry overnight and then blocked in ‘blot buffer’ (2% non-fat dry milk, 0.1% Tween-20, 50 mM NaCl, 10 mM Hepes, pH 7.4) for 30 min at room temperature. The arrays were then overlaid with control GST, GST-MapC50 or GST-NleH1C150 fusion proteins (100 nM in blot buffer) overnight at 4°C. The overlaid arrays were washed three times for five minutes each with 20 ml blot buffer, incubated with anti-GST horseradish peroxidase (HRP)-conjugated antibody (Amersham, 1:4000) for 1 h at room temperature, washed again three times for 5 min each with 20 ml blot buffer, and ultimately visualized via chemiluminescence with the ECL kit from Pierce.

### Yeast two-hybrid

Yeast two-hybrid screen using Map as prey and cDNA library as bait was performed as described before (Simpson *et al*., 2006).

### Western and far Western blot

Far Western blot was performed using 10 µg ml^−1^ of His-NHERF2, His-EspI, MBP-Map or GST-NleH1 in Tris buffered saline (TBS) 0.1% Tween20 with 1% skimmed milk followed by Western blot detection as previously described (Simpson *et al*., 2006). Western blots were performed following standard methods using rabbit anti-NHERF2 (raised against purified human His-NHERF2, Covalab), anti-EspI (Lee *et al*., 2008), anti-NleH (raised against purified His-NleH1, Covalab), mouse anti-Map (Immune Systems limited), anti-poly-Histidine (Sigma), anti-GST (AbCam) and anti-HA HRP-conjugated (Roche) antibodies. Goat anti-mouse and anti-rabbit IgG HRP conjugates (Jackson laboratories) were used as secondary antibodies.

### Transfection and generation of NHERF2 stable cell line

HeLa cells were routinely maintained in DMEM containing 10% fetal calf serum (FCS) and 1 mM l-glutamine (Gibco) in a humidified atmosphere of 5% CO_2_ at 37°C. Transfection with pBKCMV, pRK5 and pEGFP vectors and their derivatives was performed using lipofectamin 2000 (Invitrogen) according to the manufacturer's recommendations.

To generate the stable cell line, HeLa cells were grown to confluence in 75-cm^2^ cell culture flasks and transfected with pBKCMV-HA-NHERF2 using lipofectamin 2000 (Invitrogen) as previously described (Oh *et al*., 2004). After 16 h, cells were trypsinized and 2 × 10^5^ cells were sub-cultured in 100 mm Petri dishes in DMEM media supplemented with 800 µg ml^−1^ geneticin (Invitrogen). Cells were cultured for 10 days in the same Petri dish with repeated change of media for selection of resistant clones. Single clones were transferred to 96-well plates (Nalgene) and grown to confluence. Clones were then transferred to 24-well plates and replicate 24-well plates containing coverslips for expression analysis by immunofluorescence microscopy. Positive clones were trypsinized, sub-cultured in 25 cm^2^ flasks and then stored at −80°C in DMEM supplemented with 10% dimethylsulfoxide (DMSO). In this article, the stable cell line is referred as HeLa–NHERF2.

### Infection of HeLa cells

Cells were seeded onto glass coverslips 48 h prior to infection at a 70% confluence and maintained in DMEM supplemented with 10% FCS at 37°C in 5% CO_2_. Three hours before infection, cells were washed three times with PBS and the media replaced with fresh DMEM without FCS. To prime bacteria for infection, DMEM was inoculated with overnight cultures at a dilution of 1:50 of the appropriate bacteria as previously described (Collington *et al*., 1998). Recombinant protein expression was induced by addition of 1 mM of IPTG 30 min prior to infection. Infections were carried out at 37°C in 5% CO_2_ at a multiplicity of infection (MOI) of 100 for each time point (15, 30, 60 or 90 min).

### Co-Immunoprecipitation (Co-IP)

HeLa–NHERF2 cells were grown to confluence in 25 cm^2^ cell culture flasks and infected for 1 h as described above. Cells were washed 3 times with PBS and lysed in 500 µl of Co-IP buffer [50 mM Tris-HCl, pH 7.5, 100 mM NaCl, 1% NP40, 0.5% sodium deoxycholate, 10% glycerol, protease inhibitors (Complete, Roche)]. The lysate was transferred to a pre-chilled eppendorf tube and centrifuged at 9200 *g* for 10 min at 4°C. The cleared lysate was transferred to a fresh pre-chilled eppendorf tube containing 15 µl of pre-equilibrated anti-HA agarose beads (Sigma) and incubated on a spinning wheel for 2 h at 4°C. The suspension was washed three times in Co-IP buffer followed by brief centrifugation at 9200 *g* at 4°C after each wash. Bound proteins were eluted using 80 µl of 100 µg ml^−1^ HA peptide (Sigma). Analysis of the samples was performed by Western blot as described before.

### Immunofluorescence staining and microscopy

Following infection or transfection, the coverslips were washed three times in phosphate-buffered saline (PBS) and fixed with 3% paraformaldehyde (PFA) for 20 min before being washed 3 more times in PBS. The fixed cells were quenched for 20 min with PBS 50 mM NH4Cl, permeabilized for 4 min in PBS 0.25% Triton X-100 and washed three times in PBS. The samples were blocked for 1 h with PBS 5% bovine serum albumin (BSA) prior to incubation with primary and secondary antibodies. The primary mouse anti-HA (Covance) and anti-GM130 (BD transduction laboratories) as well as rabbit anti-Myc (AbCam) and anti-cleaved caspase-3 antibodies (Cell Signalling Technology) were used at a dilution of 1:200. Rabbit anti-O127 (kindly provided by Dr Roberto La Ragione, Veterinary Laboratory Agency, UK) was used at a dilution of 1:500. The samples were incubated with the primary antibody for 1 h, washed three times in PBS and incubated with the secondary antibodies for 1 h. Donkey anti-rabbit IgG conjugated to a Cy3, Cy5 or AMCA fluorophore and donkey anti-mouse IgG conjugated to Cy2, Cy3 or Cy5 fluorophores (Jackson ImmunoResearch) were used at a 1:200 dilution. Mouse anti-Myc FITC-conjugated antibody (Sigma) was used at a 1:100 dilution. Actin was stained using Rhodamine phalloidin (Invitrogen) at a 1:500 dilution and DNA was stained using Hoechst 33342 (Molecular Probes). All dilutions were prepared in PBS containing 5% BSA. Following three washes in PBS, coverslips were mounted on SuperFrost glass slides using Prolong Gold antifade reagent (Invitrogen) and visualized with a Zeiss Axioimager immunofluorescence microscope. All images were analysed using the Axiovision Rel 4.5 software.

### Apoptosis assay

For apoptosis studies, 1 µM staurosporine (STS) (Calbiochem) was added to the media during 4 h and apoptotic cells (stained either for nuclear condensation or caspase-3 activation) were counted using immunofluorescence microscopy as described (Hemrajani *et al*., 2010).

### Scanning electron microscopy (SEM)

HeLa and HeLa–NHERF2 cells transfected with pRK5-Map or pRK5-MapΔC3 were washed three times with PBS and fixed with 3% glutaraldehyde in PBS. The samples were then washed with PBS three times before being post fixed in 1% Osmium Tetroxide for 1 h. Following three washes in PBS and 15 min dehydration in graded ethanol solutions (50% to 100%), the samples were transferred to an Emitech K850 Critical Point drier and processed according to the manufacturer's instructions. The samples were coated with gold/palladium using an Emitech Sc7620 minisputter to a thickness of approximately 370 Å and examined at an accelerating voltage of 20 kV using a Jeol JSM-6390 electronic microscope.

### Statistical analysis

Results are expressed as mean values ± standard deviation. All statistical tests were performed using the program Sigma Plot version 11.0 using the parametric Student's *t*-test. *P* < 0.05 was considered as significant.

## Supporting information

**Fig. S1.** Fluorescence microscopy of HeLa–NHERF2 cells transfected with pRK5 (Mock) and pRK5-map. Mitochondria were detected with Mitotracker (red) and Map was detected with anti-myc (green). Map colocalizes with the mitochondria. Scale bar = 10 µm.

**Fig. S2.** Fluorescence microscopy of HeLa–NHERF2 cells transfected with pRK5 (Mock), pRK5-mapΔC3, pEGFP-C2-espIΔC7 and pRK5-nleH1ΔC4. NHERF2 was detected with anti-HA (green) and effectors Map and NleH1 were stained with anti-myc (red). Green GFP signal was converted to red for EspI. Neither effector colocalized with NHERF2. Scale bar = 10 µm.
