# Supplementary figures and images for: Binding to Na+/H+ exchanger regulatory factor 2 (NHERF2) affects trafficking and function of the enteropathogenic Escherichia coli type III secretion system effectors Map, EspI and NleH
Source: Cell Microbiol. 2010 Aug 3;12(12):1718–31. doi: 10.1111/j.1462-5822.2010.01503.x (PMC3015060; doi:10.1111/j.1462-5822.2010.01503.x)

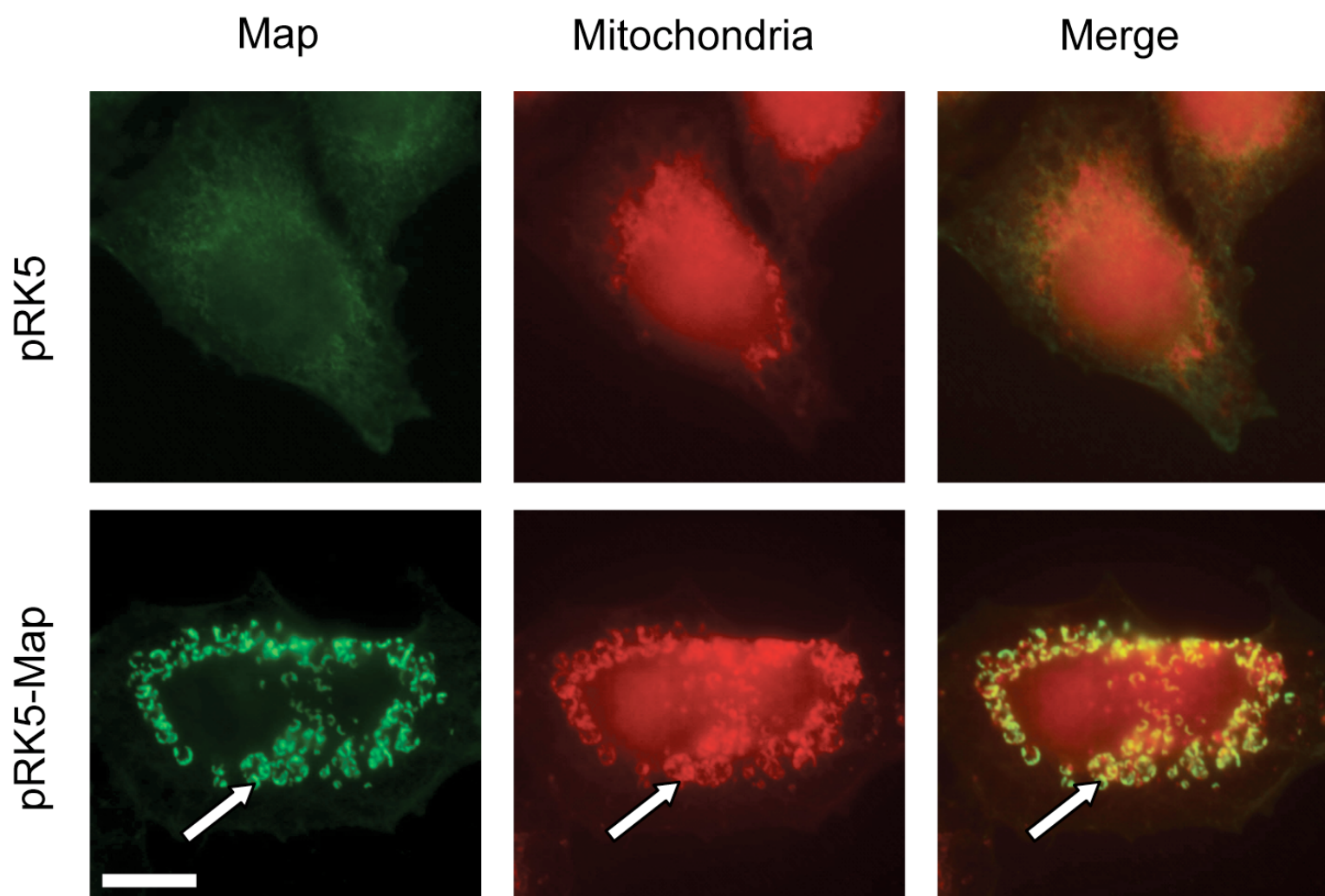

Figure S1

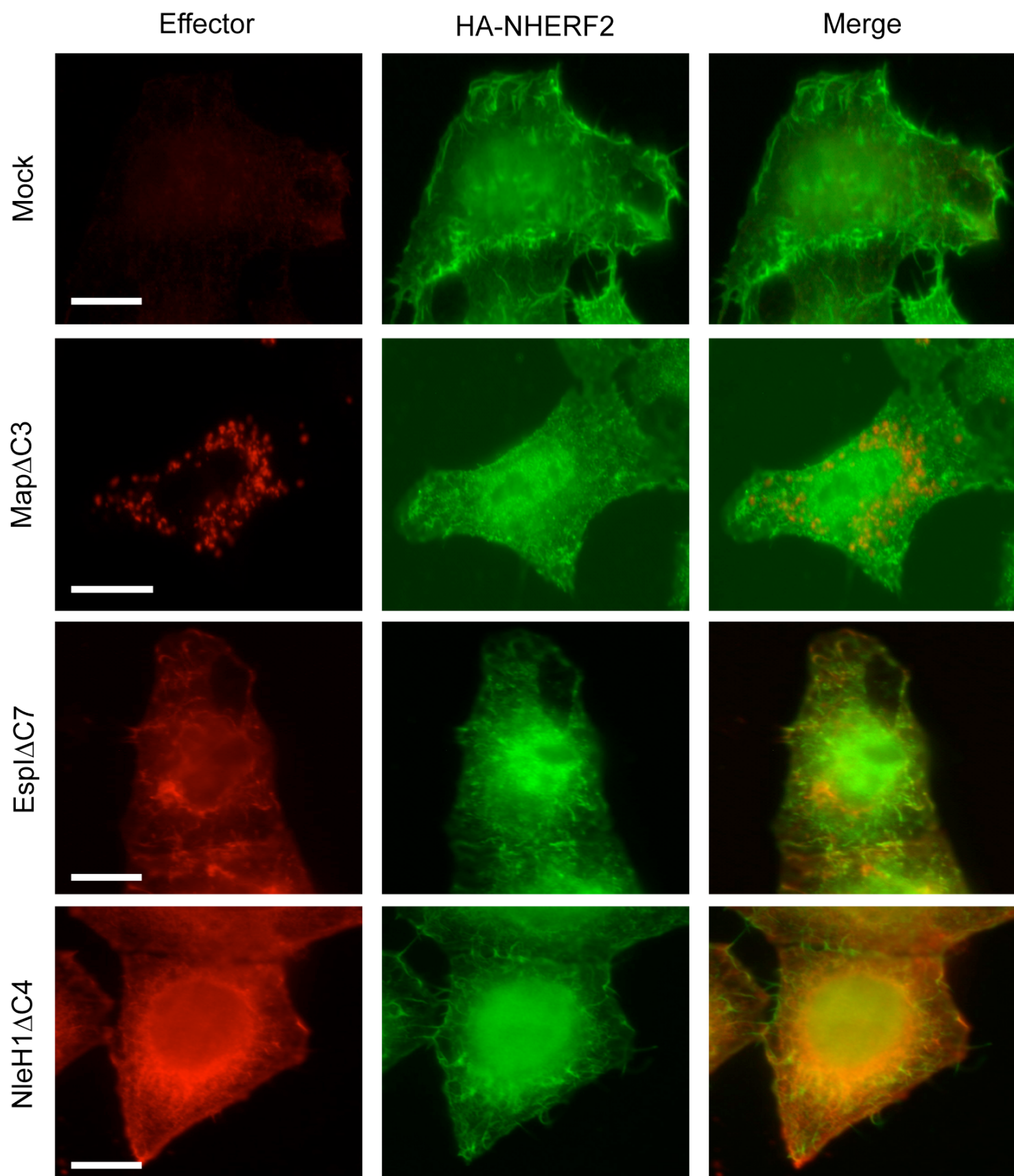

Figure S2

Supplement: Supplementary file 1 [file cmi0012-1718-SD1.pdf]
